# Aesthetically Designing Video-Call Technology With Care Home Residents: A Focus Group Study

**DOI:** 10.3389/fpsyg.2021.540048

**Published:** 2021-02-12

**Authors:** Sonam Zamir, Felicity Allman, Catherine Hagan Hennessy, Adrian Haffner Taylor, Ray Brian Jones

**Affiliations:** ^1^School of Nursing and Midwifery, University of Plymouth, Plymouth, United Kingdom; ^2^School of Medical Education, Faculty of Medical Sciences, Newcastle University, Newcastle upon Tyne, United Kingdom; ^3^Faculty of Social Sciences, University of Stirling, Stirling, United Kingdom; ^4^Peninsula Schools of Medicine and Dentistry, University of Plymouth, Plymouth, United Kingdom

**Keywords:** video-calls, focus group, design, older people, care-setting, personalization, dementia, Skype

## Abstract

**Background:**

Video-calls have proven to be useful for older care home residents in improving socialization and reducing loneliness. Nonetheless, to facilitate the acceptability and usability of a new technological intervention, especially among people with dementia, there is a need for user-led design improvements. The current study conducted focus groups with an embedded activity with older people to allow for a person-centered design of a video-call intervention.

**Methods:**

Twenty-eight residents across four care homes in the South West of England participated in focus groups to aesthetically personalize and ‘dress-up’ the equipment used in a video-call intervention. Each care home was provided with a ‘Skype on Wheels’ (SoW) device, a wheelable ‘chassis’ comprising an iPad or tablet for access to Skype, and a telephone handset. During the focus group, residents were encouraged to participate in an activity using colorful materials to ‘dress-up’ SoW. Comments before, during and after the ‘dress up’ activity were audio recorded. Framework analysis was used to analyze the focus group data.

**Results:**

Older people, including seven with dementia were able to interact with and implement design changes to SoW through aesthetic personalization. Themes arising from the data included estrangement, anthropomorphism, reminiscence, personalization, need for socialization versus fear of socialization and attitudes toward technology. After this brief exposure to SoW, residents expressed the likelihood of using video-calls for socialization in the future.

**Conclusion:**

Care home residents enjoy engaging with new technologies when given the opportunity to interact with it, to personalize it and to understand its purpose. Low cost aesthetic personalization of technologies can improve their acceptability, usability, and implementation within complex care environments.

## Introduction

Those engaged in e-health development and implementation have reported key reasons why we should include older users in research pertinent to interventions reliant on technology (Seale et al., 2002). First, they maintain that involvement can avoid the application of technology that may create more problems than it actually solves (Graafmans, 1998b). Second, their contribution in the earliest stages of research and development ‘anchors’ the technology in end-users’ views and experiences (Stern, 1994). Third, the ‘design for all’ notion claims that what works for older people will work for everyone (Sandhu, 1998). Examples of the effectiveness of older user involvement exclusive to product development are increasing within the United Kingdom (UK). The Royal Society (UK) has actively promoted the idea of older users being involved in research at the early stages of design development through the ‘New Design for Old’ project (Hewer and Kingsland, 1998). Similarly, the Centre for Applied Gerontology in Birmingham (UK) is recognized as pioneering the involvement of older people in the design and evaluation of products, forming a consumer panel of ‘1,000 elders’ (Graafmans, 1998a).

Contemporary socialization interventions for older adults incorporate internet use including applications such as Facebook (Jung and Sundar, 2016), and email (Osman et al., 2005; Cotten et al., 2013). More recently, advanced telepresence technologies that integrate video-calls have been developed, and tested among older people with and without dementia to reduce loneliness (Moyle et al., 2017). These current socialization interventions have been demonstrated to be enjoyable and beneficial for older adults who live alone or in care environments (Morris et al., 2014; Zamir et al., 2018). For example, low-cost videophones have been tested for feasibility among older, frail care home residents to enhance communication with distant relatives proving to be an effective socialization tool (Mickus and Luz, 2002). The concept of videophones has also been trialed for usability among older people with dementia and their social contacts. Similarly, the results revealed positive attitudes toward them, demonstrating users’ perceptions of videophones as worthwhile (Boman et al., 2014). Nonetheless, such studies have identified technical and design problems with an older cohort, which need to be addressed before being readily implemented as a long-term technology solution (Zamir et al., 2018).

Successful technology implementation is now more often being characterized as ‘bricolage’ (pragmatic customization of technologies), by the participant or by ‘bricolers’, someone close to them (Greenhalgh et al., 2013). The concept was first put forward by Greenhalgh et al. (2013) in relation to assistive technologies. As the world now accesses technology on a daily basis, we habitually engage in bricolage every day. We tend to put together available objects and technology devices that are at our disposal in different ways to their intended purpose to create solutions for either our social, health or mental well-being needs. For example, carriers or those with dementia engage in bricolage as they adapt assistive technologies in dynamic and innovative ways such as sticking tapes over buttons or even building their own telecare systems to meet their needs (Gibson et al., 2018).

Such ideas are being implemented in practice where residents in care homes have been able to ‘dress up’ and ‘pimp’ their Zimmer frames and other assistive objects (Health Foundation, 2017). Aesthetic personalization of technologies has been explored with consumer electronics, where design companies have realized consumers’ perceptions can sway the process of choosing and using such products (Tzou and Lu, 2009). Tzou and Lu explored the emotional (brand attachment and uniqueness), aesthetica (pleasure and beauty) and ergonomic (perceived usefulness and ease of use) conceptions that can impact public acceptance of products. Their study suggested that aesthetic facets are a vital determinant to acceptance intention.

For a successful and efficacious design development process with older adults using the idea of bricolage (Gibson et al., 2018) and personalization through shared group activities can promote a better understanding of perceptions of design features. In turn this produces outcomes that are useful for the investigators at the early stages of the research cycle (Seale et al., 2002). Therefore, focus groups or group market research activities have been advocated in health and technology advancement to allow for exploratory research where little is known in the earliest phases, or to add further depth to and understanding of the topic (Kitzinger, 1995; Seale et al., 2002).

The European project ACTION (Assisting Carers using Telematic Interventions to Meet Older people’s Needs) is one illustration of how focus groups have been applied to the topic of technology solutions. Discussions with participants revealed older people’s concerns with technology, but also the belief that modern technology could have a positive impact on their lives and well-being (Magnusson et al., 1998).

Avis et al. (2015) reported challenges and opportunities that focus groups aimed at refining digital technologies might present. These challenges, especially when including older people, can produce a long list of concerns. Aging participants may be inexperienced in using modern technologies (Vaportzis et al., 2017). Participants with dementia are not always included in such discussions meaning their views can go underrepresented (Fukuda et al., 2015; Zamir et al., 2018). Participants may be reliant on a caregiver to be present and so their responses may not always be representative. Also, older individuals may feel inadequate to contribute toward the refinement of advanced technologies, feeling it is not relevant to them (Stern, 1994; Vaportzis et al., 2017). It can be nearly impossible to control for all of the potential challenges listed above, however, focus groups with older participants are rewarding in facilitating intervention implementation and evaluation for a number of research studies (Seale et al., 2002; Demiris et al., 2004; Mitzner et al., 2010; Vaportzis et al., 2017).

**FIGURE 1:**
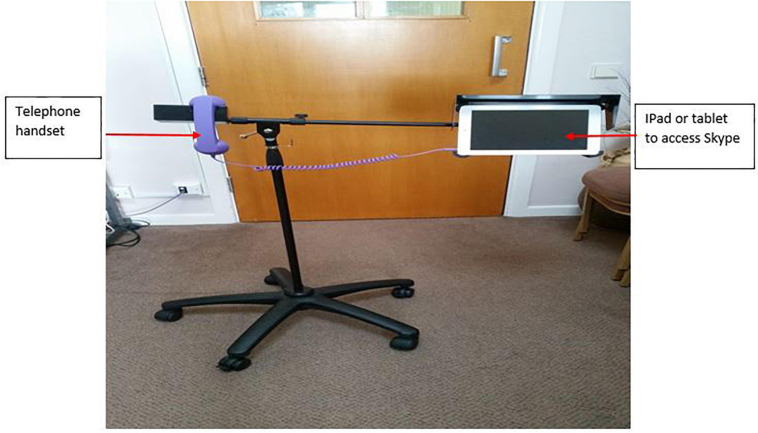
Skype on wheels (SoW) intervention.

The current study employed focus groups to address a principal barrier toward implementation of a video-call intervention, ‘Skype on Wheels’ (SoW) (Figure 1) in care homes for residents with and without dementia. The ‘Skype on Wheels’ idea in the form of telepresence robot (e.g., Giraff) had been trialed before (Tsui et al., 2011; Do et al., 2012) but was expensive. Our SoW device was intended as a ‘budget version’ with no robotics, moved around the care home by staff. The long-term aim of SoW was to improve socialization and reduce loneliness by helping residents to connect to distant loved ones and create new social contacts. Feedback from care home staff and residents in the previous research (Zamir et al., 2018) revealed key barriers to and benefits of video-calls for socialization suggesting the need for residents to aesthetically personalize or ‘dress-up’ SoW which, at that time, appeared ‘scary’ and ‘clinical looking.’

The aims of the focus groups were twofold. First, it was to elicit and qualitatively explore the views and expressions of residents toward SoW and its overall design. Second, it was to serve as an activity to allow residents to aesthetically personalize SoW, thus taking from the concept of bricolage. This focus group activity could help normalize a new technology within a complex care environment and help inform better ways to implement video-calls for socialization purposes.

## Materials and Methods

### Design

The study followed Avis et al. (2015) seven-step approach to using focus groups for refining digital technologies: (i) a ‘checklist to plan, track and report’ all aspects of the focus group; (ii) the inclusion of a ‘helper’ to act as facilitator to help manage the group dynamics and flow of the discussion; (iii) factoring in time for ‘constructive feedback’ such as unexpected remarks and negative expressions; (iv) tailoring questions to participants by moderating questions and making use of probes or prompts; (v) seeking participants’ views on elements such as aesthetics, ease and logic of navigation; (vi) managing feedback to the task at hand; (vii) leveraging the digital expert, the person with experience of the intervention, design or technology.

### Ethics

This study was approved by the University of Plymouth ethics committee in August 2016. All residents and staff gave written consent and an information sheet was provided. For those who had dementia and/or were unable to give written consent, verbal consent was gained before the focus group and a care staff member confirmed this in writing with the first author.

### Care Homes

The recruitment and participation of older people was via a convenience sample of four care homes. The criteria for inclusion of the care homes was that the managers agreed to participate and trial the use of the SoW device, they said the home had a good internet connection, and the homes were within traveling distance for the lead researcher. Characteristics of each care home including average number of residents, type of care provided, video-call equipment available and WiFi connections were noted in the study and are described below (Table 1).

**TABLE 1:** Characteristics of care homes participating.

	C1	C2	C3	C4
Number of care staff at site	45	60	15	45
Care staff participating	2	3	3	2
Average no. elderly persons in care*	30	30	17	30
Minimum age of elderly persons	65+	65+	70+	65+
Type of care given	Dementia	Dementia	Dementia	Dementia
Weekly visits**	40%	30%	95%	40%
No visits***	15%	15%	1%	10%
Video-call equipment available	• iPad • Samsung Galaxy tablet • SoW device • Telephone handset	• iPad • SoW device • Telephone handset	• iPad • SoW device • Telephone handset	• iPad • SoW device • Telephone handset
WiFi connection	Throughout the site	Throughout the site	Throughout the site	Throughout the site
Speed of WiFi* (as reported by care staff)	Good enough	Fast	Good enough	Good enough

*Between March–December 2016 **Estimated proportion of older people who were usually visited each week by loved one. ***Estimated proportion of older people who usually received no visits over a 4 weeks period.

### Participants

The participants comprised a convenience sample of 28/107 older people from the four care homes. Initially care staff identified potential participants who had ability to consent to the study (the only inclusion criterion). For those who were able to consent the first author then visited and invited them to participate to the study, regardless of physical or mental limitations or previous use of video-calls. The four focus groups ranged from five to nine participants per group. There were six males and 22 females. Ages ranged from 65 to 97 years (mean = 80 years). All participants spoke English as their first language. Race and ethnicity were not diverse within the sample; all participants were white Caucasian. Three participants had previously used video-calls but 25 had not. Eight participants were thought by the care home staff to have dementia of varying degree, two with advanced dementia (but still able to give consent), three with moderate dementia, and three with early stage dementia. Other impairments of participants were also noted. These included hearing impaired (*n* = 14), visually impaired but able to see with glasses or moving objects close enough (*n* = 9) and frailty (*n* = 10). Two participants were non-verbal but were able to lip read and communicate through sign language or gestures and from support of the care staff facilitator.

Eight care home staff took part in the study. Five care home staff participated as ‘active facilitators’ who supported the researcher in presenting SoW to residents, and supported non-verbal residents or those with dementia to partake. Three care home staff and one student from the University of Plymouth were involved as ‘inactive facilitators’ who observed interactions and made notes throughout the focus groups to improve the accuracy of data.

### Materials

**FIGURE 2:**
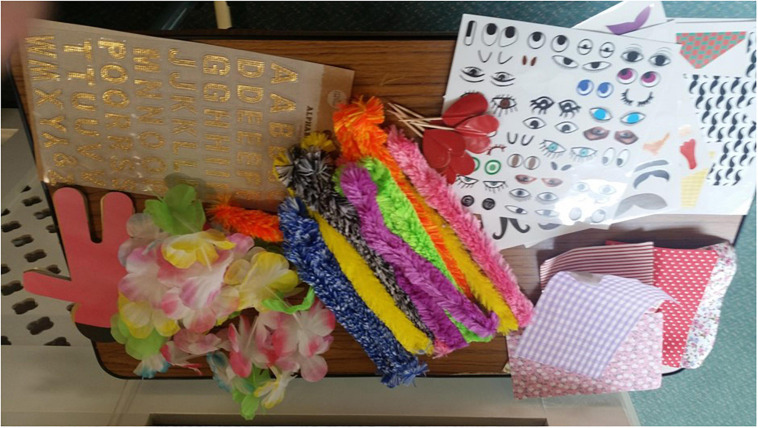
Focus group materials.

The SoW intervention comprised an iPad and a colorful telephone handset (Figure 1). Care home staff suggested materials to ‘dress-up’ and personalize SoW to improve its acceptability among residents. Specifically, three care home staff suggested the need for colorful and soft materials that residents were able to touch, feel and add on to SoW to reduce its ‘clinical’ appearance. This was similar to the art therapy sessions in two of the care homes that residents were accustomed to and enjoyed, particularly as a group activity. Materials were selected by the researcher (first author) and shown to care home staff before the commencement of the study. The same materials were used across all four focus groups, namely: stickers [letters and numbers, a sticking chalk board (A5 size), cocktail heart and star shapes], purple butterfly wings and wand, Hawaii flower necklace, bow tie, squares of different colorful tissue, small paper men and women, A4 sized colorful windmills, fluffy colorful and flexible pipes (Figure 2).

### Focus Group Script

The script was semi-structured and designed to facilitate discussion between residents regarding domains of purpose, design, and overall aesthetic appeal. In addition, the likelihood of using a telecommunication technology such as SoW for socialization was discussed. Although some residents had experience of using video-calls on a tablet or iPad, SoW was a novel device not seen by many prior to the focus groups. In previous research (Zamir et al., 2018), patients and residents were presented with SoW and reactions were recorded. Older people mostly asked “*what is this?*” and therefore our first question in the focus group was “*Do you know what this is meant to be used for?*”, which was followed by discussion prompts that varied across each group. The researcher or care home staff member who was an ‘active facilitator’ then explained SoW’s purpose and asked if participants felt the device mirrored its function. For the design domain, participants were asked “*What do you think of this device?*”, “*What do you like/dislike about this device? Why?*”, “*What would you change? How?*”, “*What would you keep the same?*”, and “*What color handset would you prefer?*” For the usability domain, participants were asked “*Do you feel comfortable using this?*” and “*Does the handset feel comfortable to you?*”, which acted as a prompt for participants to touch and feel the device.

A second discussion after ‘dressing up’ SoW was to understand whether participants felt the device was now more acceptable and normalized to their environment. This open and unstructured conversation was dependent on how each group had aesthetically personalized the device. The researcher asked each group if they wanted to participate in future video-call activities, and whether they better understood what an iPad and Skype was before the close of the focus group activity.

### Procedure

Each focus group was conducted in the care home lounge of the participating site and lasted approximately for 1 h. The researcher summarized the purpose of the focus group as being part of the University of Plymouth’s research on improving the design of new technologies for older people, and the need to gather some useful feedback from them to implement these design changes that would increase their usability. Participants were told that the technology in front of them (SoW) was a new device and was for their care home to keep, therefore it could be useful for them to personalize it to their liking. The researcher or care staff further explained the rules of the discussion (one person to speak at a time to contribute their thoughts and ideas).

Each group discussed SoW over three domains of understanding the purpose, design, and usability over two discussion points, which were at the start and end of the session. The focus group sessions were split across three segments. First, participants discussed each domain prior to ‘dressing up’ SoW. At this point, the researcher or ‘active facilitator’ wheeled the device to each participant for them to gain a closer look and feel of Sow and to further ask questions about it or make comments on its texture or features. Then, participants were given time to select and aesthetically individualize or ‘dress up’ SoW according to their personal taste with support from the researcher or ‘active facilitator’ (i.e., to physically stick on materials and move the device across to each participant). Third, participants re-discussed each domain and were asked if they wanted to participate in future video-call sessions using SoW. Throughout the focus groups, the ‘inactive facilitator’ made observations and took notes on interactions with SoW, and between participants.

### Data Collection and Analysis

The focus groups were audio recorded and for those participants who were non-verbal, the researcher described aloud the hand gestures or movements. Additionally, the ‘active facilitator’ voiced the participant’s answer to ensure the audio recording device captured all comments. Similarly, for those participants who had dementia or were unable to speak loud enough (due to frailty), the researcher repeated back what the participant had said to improve clarity and accuracy when transcribing the data. Focus groups were transcribed verbatim and personal identifying information was omitted. Observations throughout were taken as handwritten notes by the ‘inactive facilitator’ and became field note data.

Transcripts were analyzed using framework analysis as developed by Ritchie et al. (2003). Gale et al. (2013) provide a clear and comprehensive step-by-step guide in using the framework in health care research. Their outlined procedure for the analysis of the current focus group transcripts was applied. First, transcription of the audio recording was done verbatim. The researcher then became familiarized with the transcript and the observation notes were included to help interpret the data. After familiarization, open coding on the first 2–3 transcripts was done by adding a ‘label’ or paraphrase. Codes included behaviors, values, and emotions. A second researcher (second author) independently coded three (of four) focus group transcripts, and then the researcher (first author) added codes to these. Researcher one and two then developed an analytical framework by comparing the codes they had applied and agreed on a final set of codes to use. Codes were listed and grouped together into categories (if necessary) into Excel, which would become the final codes. These final codes were applied to the subsequent transcripts (including field notes from observations). Codes or categories were assigned abbreviations for easy identification in the subsequent transcripts. The analyzed data was then charted into a framework matrix, which included reducing and summarizing the data by category or code, and adding a supporting reference to each. Finally, analysis of the matrix generated themes by making connections between the codes and categories. All authors agreed the final set of themes within the manuscript.

## Results

**FIGURE 3:**
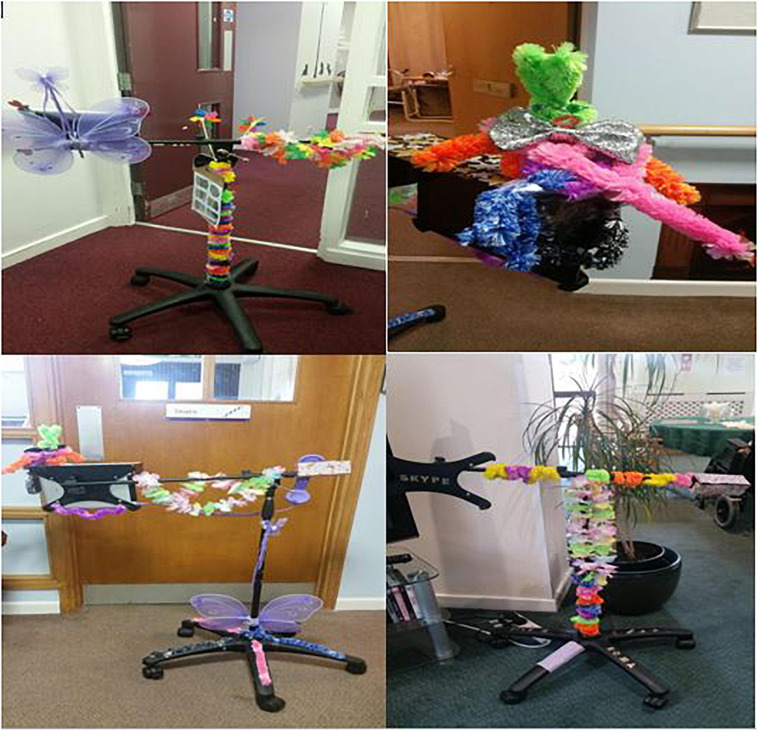
Skype on wheels dressed up.

All four care homes successfully engaged in the activity producing a noticeably distinct SoW at the end of the session (Figure 3). The analysis of the focus group data revealed codes and categories, which informed six final themes (Table 2). Residents from C1 had mixed reactions toward SoW during the session with one resident who was disinterested throughout the focus group. Here, residents preferred to interact with SoW by touching and feeling the device to understand it better. Residents from C2 appeared to be the most dismissive group pre dress-up. They portrayed more negative reactions and confusion toward SoW compared to the other care homes. This group engaged in far more talk about the appearance of the device and its aesthetic appeal, rather than the feel of it. Residents from C3 reinforced the notion of ‘personalization’ that emerged from the data. Here, residents preferred materials such as the letters and numbers to help remind them what SoW was, and to attach their personal names to the device to increase its acceptability. Residents in C3 were not confident in engaging with technology but were open to the idea of using SoW for communication with distant relatives. Residents from C4 appeared more intrigued toward the prospects of having a new technology in their home. Because of this, they focused their attention on, and selected materials that could personalize SoW to their liking.

**TABLE 2:** Final themes with corresponding categories and codes.

Themes	Categories	Codes
1-Estrangement	Obfuscated	*Uncertainty Dismissive*
2-Reminiscence	Recognizable props	*Triggered memories Initiate interest*
3-Attitudes toward technology	Expectations of technology	*Untrusting technology Aging stigma Prefer what they know Purposeful design Usefulness Activity orientated Age appropriate*
4-Anthropomorphism	Humanized	*Fables Attach names*
5-Personalization	Acceptability and usability	*Aesthetic simplicity Attractive design Adaptable Sensory design*
6-Need for socialization vs. fear of socialization	Social presence	*Peer support Hide reality Inter-socialization*

### Estrangement

Residents initially expressed negative feelings toward the SoW design, and overall technology use before dressing up the device. As a result, a theme of ‘estrangement’ emerged from the data where residents were dismissive of SoW when it was first introduced stating that they “*wouldn’t really bother with it*,” and would “*leave it for other people*” as “*it’s nothing to do with me.*” For a few, the device was noticeable which sparked interest as some residents stared at the device and pointed to it stating “*I think this would be interesting*” and remarking ‘*Oh my gosh…interesting.*” One resident from C2 found the device to be strange however, this did not deter him from wanting to use it, “*Yes I don’t mind using it…strange…but I don’t mind.*” Conversely, other residents appeared less engaged as they turned away from the device and the group, or presented signs of uncertainty when first noticing the device, as they were unable to recognize it and so were unsure of its purpose. One resident with cognitive decline was especially dismissive expressing annoyance when first seeing the device, “*I get annoyed*” but explained it is because “*I don’t know anything about it.*” Furthermore, some residents felt the nature and purpose of the device, as with most new technology, was obfuscated and needed to know more about SoW before engaging with it, or even having it in and around their environment.

“*I haven’t got a clue, because it’s strange looking maybe because all these new things are…the way they are made… we wouldn’t know what it is intended for or what to use it for round here.*” [Obfuscated- Uncertainty] (Resident, C4)

### Reminiscence

The SoW props such as the telephone handset acted as a recognizable prop. This was evident when asked what residents perceived the device was used for, as many were able to answer ‘*to make telephone calls’* or *‘to speak to people with.’* Furthermore, the shape of SoW was useful in triggering memories for some residents. One resident from C2 felt the device was similar to those that were used to take photographs during their time. Another resident from C2 similarly made comparisons stating, “*Well that’s what made me think it looks like a camera*,” with two residents from C4 who corroborated this idea.

One female resident in C2 correspondingly linked the SoW design to a telephone, specifically the old cord telephones she used to have in her home. Another fellow resident claimed it looked similar to the red public telephone booths further supporting this idea. This sparked a conversation among the group of residents in C2 who began to reminisce, and in turn initiated interest toward participating in future video-call activities. Two residents from C3 further suggested the design of SoW should mimic the famous red telephone booths (as seen in London) as they tend to be more recognizable to their age group.

“*Well I think it reminds me of almost being like the telephone on the walls you know…the red booths…so you could have that fixed on the wall and ‘telephone’ written on the side of it or probably the other way round but that’s what I think.*” [Recognizable- Triggered memories] (Resident, C3)

After the dress-up of the device and learning that the video-call app Skype is part of SoW, one resident remembered what an iPad was linking it back to SoW. The interaction between the resident and SoW triggered memories of previous encounters of similar technologies.

“*SKYPEEE…Oh OK sorry for interrupting so is that…I think I can remember now…something miniature that you carry around and write on? No that’s a different thing? But you use that for the Skype…yes*”. [Recognizable- Triggered memories] (Resident, C3)

### Attitudes Toward Technology

Residents’ body language toward SoW reflected the type of attitudes they held. For example, some residents displayed smiles, laughter, excitement and leaned forward, whereas others turned away even after it was explained what the purpose of SoW was. It appeared that residents had set expectations of technology or schemas based on previous experiences that shaped the way they perceived SoW. Many appeared untrusting of technology as residents repeatedly said ‘*No, no’* and shook their heads at the thought of using SoW for conversations. Two residents felt uncomfortable with the idea of their images being available for others to see in the screen and insisted in knowing how easy it was for the public to access their images. One resident from C2 associated SoW to a spying device “*I don’t know…I just don’t know…it’s to spy!*”. Others appeared to be untrusting of the materials used that formed the actual device (the poles), and felt that it would easily “*break apart as most of these new technologies do.*”

Much of the adverse attitudes toward technology was reflected in the comments made by some residents who clearly just prefer what they know already. One resident in particular from C2 explained that if she was given the opportunity she would have her old phone to use rather than new advanced phones. Similarly, another two residents from C4 agreed that they preferred using technologies that they were familiar with, as they felt more “*confident in using what’s always been used.*”

Attitudes toward residents’ technology use was also evident among care staff who participated as facilitators. Some care staff appeared more enthusiastic about residents engaging with SoW and were encouraging interactions through words such as “*don’t be scared of it*,” “*you might enjoy it just give it a try*,” and “*this can be fun for you.*” Nonetheless, there was an underlying, but clearly unintentional, ageist stigma attached to residents being able to engage with technology from some care staff. Care staff believed that residents would not be able to understand or be able to interact with SoW because they were unable to use other technologies such as mobile phones. This belief remained even after care staff were able to witness that residents were engaging well with SoW.

Many of the residents did not know what the purpose of SoW was which was difficult to ascertain simply through its appearance. One resident from C1 likened it to a mirror with the sole purpose of reflecting, “*Oh it’s some new way of putting up a mirror to reflect what’s going on in the room.*” Another resident from C1 explained, “*at the moment it’s a bit bare and unfunctional…what’s its use?…give a use.*” Similarly, residents from C2 felt SoW was something they could not use as it lacked an appropriate function, “*Well you can’t…what purpose for…can’t use for anything…useless poles.*” It appeared that because SoW did not aesthetically resemble a communication device, residents deemed the device as unsuitable and useless. The telephone handset was relatable which was important to residents as it helped them to recognize something familiar and distinguish its key feature of tele-communication, however, this clearly was not enough for all residents.

“*Well I don’t think it looks like a telephone really…it’s like what they say it’s strange looking, wouldn’t use that…what can it even be useful for?…No…that’s not what telephones do…look like…far too big can’t carry that…where to put it? It’s not connected up…I think it’s a bit useless. If you’re making a phone call…you just put that in your hand [handset] and talk…you’re not watching that you’re just listening for the sound*”. [Expectations of technology- Untrusting technology, Usefulness, Prefer what they know] (Resident, C2)

In C3, once residents had a closer look and feel of SoW, they began to understand its use. One resident at first expressed the view that the device was just an “*iron bar*,” however, when she began to handle the device she changed her outlook suggesting that small changes could improve its aesthetic purpose.

“*It feels just like an iron bar…an iron bar in the piece and that of course is just plastic. Yes this is nice and light actually [touches the handset and iPad], yes I can see it…I can see its fine it’s a wonderful thing…and I suppose link that part and being able to have it and see it [see into the iPad camera so it shows the resident’s face] would make it look better for the purpose*”. [Expectations of technology- Purposeful design, Usefulness] (Resident, C3)

Along with the need for SoW to have clear design features to highlight that its purpose is for communication, residents in C2 felt the design should also show its appropriateness for adults rather than children. One female resident during the dress-up phase expressed that the device needs to be designed in a way that its purpose is clearly apparent to be for adult use, in case children come across SoW and damage it.

“*You don’t want to make it too colorful because it’s for us over here…maybe for children…if you had it for children they would probably mess it up and pull things off, use it for something else…then the whole idea the function its purpose is gone and you start over…its look should be for us here*”. [Expectations of technology-Age appropriate, Purposeful design] (Resident, C2)

The idea that SoW should be linked to or represent an enjoyable activity was present among residents in C4. Once residents were reminded that the purpose of SoW was to act as a means of communication to connect with distant family and the public, residents became excited at the thought of this and asked if it could be a regular activity. Furthermore, the idea of engaging in activity to improve understanding of SoW and future usability was evident across all four care homes. The majority of residents did not initially understand the purpose of SoW prior to dress up, but better grasped its use after the dress up activity and were keen to continue engaging with SoW in this way. Finally, care staff from C1, C2, and C4 all mentioned that if residents, especially those with a cognitive impairment or physical disability, were able to interact with SoW through activities then it would improve their understanding of technology and increase their likeability of the device.

“*It’s clear actually that if they just interacted with this* [SoW] *in a fun way…like it is more of an activity which is fun and not some scary thing w’ere pushing onto them…you know…because then if it’s a fun activity this thing* [SoW] *it has a need for them…it’s not some random thing…I think we will see a lot more people here remember what it is and want to KEEP using. I think let’s plan this as activities.* [Expectations of technology-Activity orientated, Purposeful design] (Care staff, C4)

### Anthropomorphism

During the ‘dress-up’ phase of the focus groups, older people began to attribute humanized features and characteristics to the SoW device. Even despite the materials resembling animal and human traits (such as the butterfly wings and eyes and mouth stickers), older people used the materials in a way that the device appeared to be less of a standardized technology instrument and instead an animal or human character. Residents from C1 and C2 dressed up the device to emulate animal and human characteristics, which then developed into stories or fables. C2 residents created a story about ‘*Rupert the rabbit,’* which was artistically hand crafted by a female resident who appeared to have poor dexterity (care staff reported and observations made). Furthermore, another two residents from C2 were keen on attaching the wings to SoW as one resident told a story about a ‘flutterby’ (a butterfly) from her childhood to the group. The resident then referred to SoW as “*the flutterby that calls*,” and decided to give it a face to make it appear more ‘real.’ The remainder of the group suggested that the device would now be associated with the made up character “*Rupert Rabbit*” so they can better remember what the device was.

“*Well it’s supposed to be a man…well a rabbit and that’s a log he’s carrying…that’s its ears…I used to do a lot of patchwork so this would be useful…it’s no trouble at all really. Just twist this…its nothing too complicated to spruce it up* [SoW]*…this is my handiwork no trouble…let’s have another look of it once we stick it on there* [on top of the iPad on SoW]. *I don’t like evil looking ones. He’s a nice fluffy bunny that will sit on this making it nice to look at*”. [Humanized-Fable] (Resident, C2)

Residents from C1 used materials that represented human features such as eyes, a nose and even referred to the SoW as having feet, “*that’s for putting on the feet.*” Residents began to decorate SoW to resemble a human as they dressed the device with a bowtie and wrapped a flower necklace around its neck.

### Personalization

Each care home, and some individual residents within each focus group, preferred to dress-up SoW to suit their needs and likeability. This person-centered approach improved the acceptability and usability of SoW where residents appeared far more positive about SoW after dress-up, “*I like this…looks better now*,” “*I think we can say good morning to it* [SoW] *every time we walk past it*,” and “*OK so that’s what Skype is…yes I am keen.*” Furthermore, residents in C3 and C4 made use of the sticky letter materials to add words onto the device such as ‘Skype,’ but also their personal names. This increased a sense of personal connection to the device, with residents claiming “*now I have a personal connection to it.*”

In terms of technological design, residents had a preference for aesthetic simplicity, which they expressed would be more advantageous among their age cohort. One resident from C4 explained that “*technologies these days get too confusing to look at, I would make this look just simple…just add color…it’s better for our age.*” Additionally, a common word iterated among almost all four groups was the word ‘*neat.*’ Residents continually expressed the need for the device to look neat which can translate to simplicity. Importantly, residents with mild to moderate dementia agreed the device should look neat and simple, and not so ‘*busy.’*

Because residents were living in a care home environment, both care staff and residents believed that SoW could easily get lost or go unnoticed in a large busy setting, blending into the background. Therefore, there was a need to make the device more perceptible but with an attractive design that was agreeable to all. Residents from C2 liked the idea of decorating the device with purple colors as the care home and its care staff uniforms were purple, “*Purple is our home.*” Furthermore, residents from C1 explained that bold colors would be eye-catching making the device more interesting, yet also a useful way to remind the residents that the device is in their home.

“*Well it’s different isn’t it….looks like a fairground…very bright…attractive design. It’s far more interesting to the eye, will be able to remind us of this SKIEE is it? Oh yes…Skype*”. [Acceptability and usability-Attractive design, Aesthetic simplicity] (Resident, C1)

After dressing up SoW some residents suggested that the design should be interchangeable. Not all the residents agreed on the materials that were placed onto SoW, especially from C1 and C2, so as a group it was agreed that these materials could be changed later. Also, the device body should be adaptable for shape and size to better match the residents’ preferences.

As the focus groups progressed, residents increased their touching and feeling of SoW. They made comments on the texture such as *‘cold’* and *‘hard.’* Residents selected materials that were soft and appealing to their senses and so *sensory design* became an important indicator of person-centered design.

“*I do like, it’s like a soft brush…feels like feathers. It’s nice, lovely and soft so we can wrap this [on to SoW] going around the long bar in the middle…yes that’s nice they’re warm aren’t they…to the touch.*” [Acceptability and usability- Sensory design, Attractive design] (Resident, C3)

### Need for Socialization vs. Fear of Socialization

Two residents from C4 expressed the desire to interact with others, “*Oh so I can see other people’s faces through this like a mirror? Yes that would be delightful to see a new face*” and “*We don’t get out much because of this wheelchair I don’t see many people. It could be useful* [SoW].” Some residents in C2 and C3 were especially keen to get started with using SoW for communication so that they began to discuss where a suitable spot would be to place it in their care home, and ways to ‘dress up’ the device to make it easier to make and receive calls. Although a number of residents stated they would like to reconnect with distant relatives through SoW, some were apprehensive and worried that their relatives would not want to.

“*Oh my gosh. Oh yeah…yes…I’ve got a granddaughter yes. I could give it a go. I don’t know about her thought…maybe. They wouldn’t want to possibly.*” [Social presence-Hide reality] (Resident, C3)

In addition to stimulating the desire to connect to others through SoW, the focus group activity initiated socialization within the care home among the residents. The dress-up of SoW enabled residents to interact and work together where they normally would not have due to the lack of such group activities available to them. Some residents found the activity to be very enjoyable and saw it as a peer game.

“*Well it wouldn’t look better on anything else…so where on there? Would you like it on you* [turns to fellow resident*]? Alright OK…Where’s* [fellow resident], *do you think he will like it on him? I didn’t know you were into this sort of thing…never seen you so interested.* [Social presence-Peer support, Inter-socialization] (Resident, C2)

Alternatively, a number of residents appeared displeased with the thought that others would be able to see their faces through the iPad screen. Some residents presented signs of insecurity toward their own image, “*Well I can barely see my own face …which I don’t like*” and “*I’ve got a big nose and bump on my nose, oh I’m not good looking…I wouldn’t want anyone to see this, no*” and “*I look too fat on that and big.*” Other residents expressed they would not want to use SoW with family members because their surroundings and environment would be too revealing to others. They preferred not to have close relatives “*see into MY world.*”

## Discussion

The findings highlighted that there are negative views toward a new or unknown technology such as SoW for older people; however, after a short period of engagement older people are likely to accept the new technology. Overall, discussions about and interacting with SoW directly, improved the acceptability and usability of the device for both residents and care staff. Our study supplements previous research that has investigated older people’s attitudes and perceptions toward a broad set of new technologies (Mitzner et al., 2010). Other studies have focused on one specific technology such as tablets, and have also incorporated a more hands-on interactive element to the focus group to help participants understand the technology (Vaportzis et al., 2017). Our study gives insights, which should be taken into account when tailor making, or designing novel technology solutions aimed at an older population.

The data analyzed produced themes that are consistent with the literature, corroborating other qualitative research findings. Participants in similar studies with older adults have expressed ‘frustrations,’ ‘limitations,’ ‘usability concerns,’ and have often mentioned how technology can look and be overly complicated (Mitzner et al., 2010; Heinz et al., 2013). These themes closely relate to our theme of ‘estrangement.’ Other researchers have also noted that higher anxiety, fear, or lack of confidence in using technology results in lower use of the new technology (Czaja et al., 2006). Our findings suggest the opposite as residents who first appeared uninterested or indifferent, later and quite quickly warmed up to the idea of video-calls. This can be explained as a result of residents familiarizing themselves with SoW through direct interaction with the device, filling in gaps in their understanding of its purpose and so reducing any fears or confusion they might have. This is also consistent with other research suggesting that the perceived potential benefits are more indicative of technology acceptance than the negative perceptions that can induce fear or lack of usability. Rogers’s (2010) theory of diffusion of innovations supports this notion indicating that older adults are less likely to adopt new technologies unless they have a clear understanding of the benefits of using them.

A focus group with an embedded activity that used creative materials demonstrated the artistic skills that older people can bring toward technology design, and highlights the need for basic elements of design to begin right at the outset of implementation. The idea of person-centered designs, bricolage and collaborative working with participants is increasingly becoming the desired standard in implementation research (Zamir et al., 2018). For technology interventions, a large sum of money is spent on changing the interfaces or key features to better match the user-needs of the older person (Newell et al., 2011; Boman et al., 2012). The current study drew on low-cost materials and techniques (a simple group activity) to allow older people to personalize a new technology (becoming ‘bricolers’) rather than completely re-designing it.

Anthropomorphism is the attribution of human-like qualities and form to non-human objects (Bartneck et al., 2009). We used that term to classify the focus group’s attribution of animal or human-like characteristics to the SoW. Social robotics has an extensive literature on this topic distinguishing between biomorphic [devices with features of biological origin, such as animal ears or noses (Klamer and Allouch, 2010)], zoomorphic [devices completely identifiable as a known animal (Moyle et al., 2019)], and anthropomorphic. Our study reinforces other work that biomorphic, zoomorphic, and anthropomorphic characteristics are likely to improve their acceptability and possible usability.

A key theme of reminiscence came through in the dataset. Not only was reminiscence useful as a means to help residents to recall technologies of their own time, but also allowed them to connect to new forms of technology on a deeper level that is personalized to their life experiences, in turn improving its acceptability and future usability. However, there is a need for follow-up studies to see examine how effective personalization was in triggering memories over a longer period.

Socialization was split across the need to engage with others, and the fear of socialization. The latter was attributed to poor self-image exhibited by some residents. Currently, there is not much literature to substantiate or validate this finding of poor self-image in relation to technology acceptance. It would be expected that poor self-image would result in not wanting to use video-calls for socialization. However, those who displayed poor self-image and so presented negative emotions toward SoW later warmed up to the idea of participating in future video-call activities. Future research should investigate whether themes of self-image are an important indicator of engaging in video-call socialization with older people.

The study included people with dementia to ensure that the research was inclusive and representative of all residents. However, upon reflection it was difficult to clearly, and effectively capture the interactions and comments of those with more moderate to advanced stages of dementia. The dynamics of a focus group are fast moving with multiple conversations and interactions that begin to overlap and so this could be a reason why this task seemed difficult. Other researchers have included people with dementia in their focus group research and have also found challenges of varying degree (Stephan et al., 2018), but that is not to say that we cannot include people with dementia in focus groups. Participation of people with dementia in this study was still incredibly valuable as it proved that they are able to, with some assistance, interact with a new technology and provide useful suggestions on its design.

The current study has further limitations that need to be acknowledged. Most participants did not have experience in using similar technologies such as an iPad or even modern mobile phones. The answers that they gave were largely based on the general views they had about technology rather than video-calls specifically. Other researchers have experienced this and suggest that although this should not be disregarded, future research should present older adults with detailed scenarios or case studies in order to further investigate the topic at hand (Mehra et al., 2016).

It can be argued, with some justification, that the bricolage approach is (sometimes literally) taking a sticking plaster to a product design that was ageist by not involving older people (including those with dementia) in the first place. Thousands of products are being used around the world by older people with sensory or other impairment with ‘added’ labels, stickers, and ‘blue tack’ to work around initial design problems. Ideally, usability studies in the development of products will identify designs that suit all potential users but we recognize that businesses will design for the market that they think will bring them profit. Our study was of a physical device (the SoW chassis); we had no control over the proprietary iPad or the Skype software. The focus group exercise served the dual purpose of gaining more immediate acceptability for the devices while indicating possibilities for future design. The bricolage approach can help achieve that in many situations but the needs of these with less consumer power require protection with legislation.

Our study was based on a convenience sample of 28 participants – about 1 in 4 residents in four care homes. Individual characteristics such as previous occupations, and levels of schooling were not documented. Our participants were mainly white caucasian women. A larger, more systematically selected sample from care homes with more diverse population may have given different results and allowed exploration of differences by demographic and clinical characteristics. Nonetheless, the methodology used demonstrates that interactive focus groups using low-cost materials to dress-up technology can be an adopted activity in all care homes.

## Conclusion

The results from this focus group study suggest that the interactive methodology employed enabled older people to describe and demonstrate what they preferred a new technology to look like. Dressing up the device using low cost materials improved residents’ understanding of what the technology was, improved the acceptability of a new technology, and increased the likelihood of the new technology being used in the near future. Further exploration of the materials does, however, need to be done to validate the idea of a humanized technology. The current focus group research was sufficient to be tasked as a step one or first activity for residents to undertake to improve intervention implementation within a complex care environment.

## Data Availability Statement

The datasets generated for this study are available on request to the corresponding author.

## Ethics Statement

The studies involving human participants were reviewed and approved by University of Plymouth. The patients/participants provided their written informed consent to participate in this study.

## Author Contributions

SZ led on the design of the project, recruitment of participants in collaboration with care staff, data collection at each site, analysis and interpretation of data, and wrote the first and final drafts of the manuscript. FA assisted with the qualitative analysis, identification of the codes and final themes, and contributed toward the critical revision of the manuscript. CH assisted with the development of the methods, identification of the qualitative analysis method, and contributed toward the critical revision of the manuscript. AT assisted with the development of the methods, and contributed toward the critical revision of the manuscript. RJ led for the project, led on the conception and design of SoW, is the supervisor for the main author SZ, and provided critical revision of the manuscript drafts. All authors read and approved the final manuscript.

## Conflict of Interest

The authors declare that the research was conducted in the absence of any commercial or financial relationships that could be construed as a potential conflict of interest.
